# Supplementary material for: How weather affects cognitive and physical outcomes in older adults
Source: PLoS One. 2025 Nov 25;20(11):e0335866. doi: 10.1371/journal.pone.0335866 (PMC12646423; doi:10.1371/journal.pone.0335866)
Supplement: S1 Table — (DOCX) [file pone.0335866.s001.docx]

**Supplementary Table 1 : Comparison of the weather per treatment arm in MAPT**

|  | Placebo | Multidomain Intervention +  Polyunsaturated fatty Acids | Polyunsaturated fatty Acids | Multidomain Intervention + Placebo | p value |
| --- | --- | --- | --- | --- | --- |
| Count (Day-Center)* | 1399 | 1407 | 1355 | 1454 |  |
| Season [Count (Percent)] | | | | | 0.709 |
| Fall | 368 (26.3%) | 399 (28.4%) | 358 (26.4%) | 406 (27.9%) |  |
| Spring | 369 (26.4%) | 356 (25.3%) | 326 (24.1%) | 349 (24.0%) |  |
| Summer | 262 (18.7%) | 243 (17.3%) | 253 (18.7%) | 263 (18.1%) |  |
| Winter | 400 (28.6%) | 409 (29.1%) | 418 (30.8%) | 436 (30.0%) |  |
| Temperature C° (Median [Quartile 1-3]) | | | | |  |
| Minimum | 8.4 [3.8;13.1] | 8.2 [3.4;12.9] | 8.1 [3.4;13.1] | 8.4 [3.7;13.2] | 0.723 |
| Mean | 12.2 [7.2;17.4] | 11.6 [6.8;16.9] | 11.5 [7.1;17.2] | 12.0 [7.3;17.6] | 0.470 |
| Maximum | 16.1 [10.6;22.1] | 15.4 [10.6;21.4] | 15.6 [10.6;21.9] | 16.1 [10.6;22.2] | 0.395 |
| Humidex (Median [Quartile 1-3]) | | | | | |
| Minimum | 8.1 [2.0;14.8] | 7.9 [1.6;14.3] | 7.8 [1.5;14.5] | 8.2 [2.1;14.6] | 0.680 |
| Mean | 12.7 [5.8;19.5] | 11.9 [5.6;18.8] | 11.7 [5.7;19.2] | 12.4 [6.0;19.6] | 0.509 |
| Maximum | 16.9 [9.7;24.1] | 15.9 [9.4;23.4] | 16.0 [9.9;23.8] | 16.8 [9.9;24.2] | 0.456 |

*Day/Center means that the unit of description is a day in a center where at least a visit happened in this treatment group. Of note this leads to more day centers than presented in table 2 as day centers are counted more than once if patients from different treatment group had a visit on the same day at the same place.
