# Supplementary material for: How weather affects cognitive and physical outcomes in older adults
Source: PLoS One. 2025 Nov 25;20(11):e0335866. doi: 10.1371/journal.pone.0335866 (PMC12646423; doi:10.1371/journal.pone.0335866)
Supplement: S2 Table — (DOCX) [file pone.0335866.s002.docx]

**Supplementary table 2: Effect of the weather on cognitive outcomes**

|  | Composite Z-score (1) | Digit Symbol Substitution Test (2) | Category Fluency (2) | Free and Cued Selective Reminding test (2), (3) | Mini-Mental State Examination (Total) | Subjective memory performance (2)(4) |
| --- | --- | --- | --- | --- | --- | --- |
| *Season (Reference Autumn)* | | | | | | |
| Spring | 1.05 CI 95% [-1.75, 3.85]  p = 0.461 | -0.232 CI 95% [-0.572, 0.109]  p = 0.183 | 0.238 CI 95% [-0.115, 0.592]  p = 0.187 | 0.306 CI 95% [-0.105, 0.717]  p = 0.144 | -0.0792 CI 95% [-0.177, 0.0183]  p = 0.111 | -0.488 CI 95% [-1.46, 0.488]  p = 0.327 |
| Summer | 1.77 CI 95% [-1.95, 5.49]  p = 0.352 | 0.094 CI 95% [-0.36, 0.548]  p = 0.685 | 0.31 CI 95% [-0.149, 0.768]  p = 0.186 | -0.639 CI 95% [-1.19, -0.0917]  p = 0.022* | -0.049 CI 95% [-0.171, 0.0731]  p = 0.432 | -0.0854 CI 95% [-1.3, 1.13]  p = 0.891 |
| Winter | 4.87 CI 95% [1.45, 8.29]  p = 0.005* | 0.085 CI 95% [-0.333, 0.503]  p = 0.69 | 0.582 CI 95% [0.161, 1]  p = 0.007* | 0.387 CI 95% [-0.116, 0.891]  p = 0.132 | 0.013 CI 95% [-0.0984, 0.124]  p = 0.819 | -1.37 CI 95% [-2.48, -0.254]  p = 0.016* |
| *Temperature °C (for 10°C)* | | | | | | |
| Minimum | -0.551 CI 95% [-2.11, 1.01]  p = 0.49 | -0.0188 CI 95% [-0.209, 0.172]  p = 0.847 | -0.0367 CI 95% [-0.235, 0.162]  p = 0.717 | -0.381 CI 95% [-0.611, -0.152]  p = 0.001* | -0.0139 CI 95% [-0.0684, 0.0407]  p = 0.618 | 0.683 CI 95% [0.134, 1.23]  p = 0.015* |
| Mean | -0.781 CI 95% [-2.25, 0.69]  p = 0.298 | -0.0501 CI 95% [-0.23, 0.129]  p = 0.584 | -0.0765 CI 95% [-0.263, 0.11]  p = 0.422 | -0.346 CI 95% [-0.562, -0.13]  p = 0.002* | -0.0145 CI 95% [-0.0658, 0.0368]  p = 0.579 | 0.522 CI 95% [0.00566, 1.04]  p = 0.048* |
| Maximum | -0.755 CI 95% [-2.06, 0.551]  p = 0.257 | -0.0516 CI 95% [-0.211, 0.108]  p = 0.525 | -0.0676 CI 95% [-0.233, 0.0981]  p = 0.424 | -0.26 CI 95% [-0.452, -0.0679]  p = 0.008* | -0.0166 CI 95% [-0.0622, 0.0289]  p = 0.474 | 0.322 CI 95% [-0.136, 0.781]  p = 0.168 |
| *Humidex (for 10 points)* | | | | | | |
| Minimum | -0.518 CI 95% [-1.68, 0.644]  p = 0.382 | -0.0104 CI 95% [-0.152, 0.131]  p = 0.885 | -0.0431 CI 95% [-0.19, 0.104]  p = 0.567 | -0.286 CI 95% [-0.457, -0.116]  p = 0.001* | -0.0103 CI 95% [-0.0508, 0.0302]  p = 0.618 | 0.522 CI 95% [0.114, 0.93]  p = 0.012* |
| Mean | -0.65 CI 95% [-1.77, 0.469]  p = 0.255 | -0.0308 CI 95% [-0.167, 0.106]  p = 0.658 | -0.0679 CI 95% [-0.21, 0.0742]  p = 0.349 | -0.264 CI 95% [-0.429, -0.1]  p = 0.002* | -0.0101 CI 95% [-0.0492, 0.0289]  p = 0.612 | 0.468 CI 95% [0.0754, 0.861]  p = 0.02* |
| Maximum | -0.702 CI 95% [-1.75, 0.347]  p = 0.19 | -0.0448 CI 95% [-0.173, 0.0831]  p = 0.492 | -0.0622 CI 95% [-0.195, 0.0709]  p = 0.36 | -0.223 CI 95% [-0.377, -0.0691]  p = 0.005* | -0.011 CI 95% [-0.0476, 0.0256]  p = 0.557 | 0.376 CI 95% [0.00731, 0.744]  p = 0.046* |

1. Z score is the mean of the Z scores of Digit Symbol Substitution Test, Category Fluency, Free and Cued Selective Reminding test and Mini-Mental State Examination (Orientation), it has been multiplied by 100 due to the small magnitude of weather effects
2. Z score multiplied by 100
3. Free and total recall
4. 1 to 100 VAS asking “How well does your memory work?”

*p value<0.05
