# Supplementary material for: How weather affects cognitive and physical outcomes in older adults
Source: PLoS One. 2025 Nov 25;20(11):e0335866. doi: 10.1371/journal.pone.0335866 (PMC12646423; doi:10.1371/journal.pone.0335866)
Supplement: S3 Table — (DOCX) [file pone.0335866.s003.docx]

**Supplementary Table 3: Effect of the weather (with 3 days lag) on cognitive outcomes**

|  | Composite Z-score (1) | Digit Symbol Substitution Test (2) | Category Fluency (2) | Free and Cued Selective Reminding test (2), (3) | Mini-Mental State Examination (Total) | Subjective memory performance (2)(4) |
| --- | --- | --- | --- | --- | --- | --- |
| *Temperature C° (for 10°C)* | | | | | | |
| Minimum | -0.847 CI 95% [-2.43, 0.734]  p = 0.294 | -0.0306 CI 95% [-0.223, 0.162]  p = 0.756 | -0.144 CI 95% [-0.35, 0.0614]  p = 0.169 | -0.384 CI 95% [-0.616, -0.152]  p = 0.001* | -0.0162 CI 95% [-0.0714, 0.0389]  p = 0.564 | 0.659 CI 95% [0.109, 1.21]  p = 0.019* |
| Mean | -0.841 CI 95% [-2.38, 0.703]  p = 0.286 | -0.0352 CI 95% [-0.224, 0.153]  p = 0.714 | -0.124 CI 95% [-0.33, 0.0817]  p = 0.237 | -0.388 CI 95% [-0.615, -0.162]  p = 0.001* | -0.0143 CI 95% [-0.0681, 0.0394]  p = 0.601 | 0.703 CI 95% [0.166, 1.24]  p = 0.01* |
| Maximum | -0.766 CI 95% [-2.1, 0.571]  p = 0.261 | -0.0514 CI 95% [-0.215, 0.112]  p = 0.537 | -0.0353 CI 95% [-0.213, 0.143]  p = 0.698 | -0.297 CI 95% [-0.494, -0.101]  p = 0.003* | -0.00278 CI 95% [-0.0493, 0.0438]  p = 0.907 | 0.444 CI 95% [-0.0218, 0.909]  p = 0.062 |
| Humidex (for 10 points) | | | | | | |
| Minimum | -0.68 CI 95% [-1.89, 0.527]  p = 0.27 | -0.0266 CI 95% [-0.174, 0.121]  p = 0.723 | -0.122 CI 95% [-0.284, 0.0394]  p = 0.138 | -0.292 CI 95% [-0.47, -0.115]  p = 0.001* | -0.0124 CI 95% [-0.0545, 0.0297]  p = 0.564 | 0.521 CI 95% [0.0996, 0.942]  p = 0.015* |
| Mean | -0.715 CI 95% [-1.89, 0.46]  p = 0.233 | -0.0258 CI 95% [-0.169, 0.118]  p = 0.724 | -0.118 CI 95% [-0.275, 0.0391]  p = 0.141 | -0.3 CI 95% [-0.473, -0.128]  p = 0.001* | -0.00933 CI 95% [-0.0503, 0.0316]  p = 0.655 | 0.572 CI 95% [0.163, 0.981]  p = 0.006* |
| Maximum | -0.671 CI 95% [-1.77, 0.424]  p = 0.23 | -0.0305 CI 95% [-0.164, 0.103]  p = 0.654 | -0.0701 CI 95% [-0.218, 0.0779]  p = 0.353 | -0.264 CI 95% [-0.424, -0.103]  p = 0.001* | -0.0031 CI 95% [-0.0412, 0.035]  p = 0.873 | 0.459 CI 95% [0.0775, 0.84]  p = 0.018* |

*p value<0.05,

1. Z score is the mean of the Z scores of Digit Symbol Substitution Test, Category Fluency, Free and Cued Selective Reminding test (2)Mini-Mental State Examination (Orientation), it has been multiplied by 100 due to the small scale of weather effects
2. Z score multiplied by 100
3. Free and total recall
4. 1 to 100 VAS asking “How well does your memory works”
