# Supplementary material for: How weather affects cognitive and physical outcomes in older adults
Source: PLoS One. 2025 Nov 25;20(11):e0335866. doi: 10.1371/journal.pone.0335866 (PMC12646423; doi:10.1371/journal.pone.0335866)
Supplement: S4 Table — (DOCX) [file pone.0335866.s004.docx]

**Supplementary Table 4: Effect of the weather (with 10 days lag) on cognitive outcomes**

|  | Composite Z-score (1) | Digit Symbol Substitution Test (2) | Category Fluency (2) | Free and Cued Selective Reminding test (2), (3) | Mini-Mental State Examination (Total) | Subjective memory performance (2)(4) |
| --- | --- | --- | --- | --- | --- | --- |
| *Temperature C° (for 10°C)* | | | | | | |
| Minimum | -1.38 CI 95% [-3, 0.235]  p = 0.094 | -0.0191 CI 95% [-0.217, 0.179]  p = 0.85 | -0.144 CI 95% [-0.35, 0.0614]  p = 0.169 | -0.452 CI 95% [-0.69, -0.214]  p = <0.001* | -0.00143 CI 95% [-0.0579, 0.055]  p = 0.96 | 0.721 CI 95% [0.159, 1.28]  p = 0.012* |
| Mean | -1.04 CI 95% [-2.66, 0.585]  p = 0.21 | -0.0203 CI 95% [-0.218, 0.178]  p = 0.84 | -0.124 CI 95% [-0.33, 0.0817]  p = 0.237 | -0.393 CI 95% [-0.631, -0.155]  p = 0.001* | -0.00219 CI 95% [-0.0586, 0.0542]  p = 0.939 | 0.764 CI 95% [0.202, 1.33]  p = 0.008* |
| Maximum | -0.559 CI 95% [-1.96, 0.843]  p = 0.434 | -0.0321 CI 95% [-0.203, 0.139]  p = 0.714 | -0.0353 CI 95% [-0.213, 0.143]  p = 0.698 | -0.324 CI 95% [-0.53, -0.118]  p = 0.002* | 0.0128 CI 95% [-0.0359, 0.0615]  p = 0.606 | 0.558 CI 95% [0.0708, 1.04]  p = 0.025* |
| *Humidex (for 10 points)* | | | | | | |
| Minimum | -1.14 CI 95% [-2.42, 0.132]  p = 0.079 | -0.0157 CI 95% [-0.171, 0.14]  p = 0.843 | -0.122 CI 95% [-0.284, 0.0394]  p = 0.138 | -0.356 CI 95% [-0.544, -0.169]  p = <0.001* | -0.00367 CI 95% [-0.0481, 0.0408]  p = 0.871 | 0.574 CI 95% [0.13, 1.02]  p = 0.011* |
| Mean | -0.869 CI 95% [-2.11, 0.369]  p = 0.169 | -0.00432 CI 95% [-0.155, 0.147]  p = 0.955 | -0.118 CI 95% [-0.275, 0.0391]  p = 0.141 | -0.308 CI 95% [-0.49, -0.126]  p = 0.001* | -0.00234 CI 95% [-0.0454, 0.0407]  p = 0.915 | 0.602 CI 95% [0.173, 1.03]  p = 0.006* |
| Maximum | -0.601 CI 95% [-1.77, 0.567]  p = 0.314 | -0.00801 CI 95% [-0.15, 0.134]  p = 0.912 | -0.0701 CI 95% [-0.218, 0.0779]  p = 0.353 | -0.295 CI 95% [-0.467, -0.124]  p = 0.001* | 0.00873 CI 95% [-0.0318, 0.0493]  p = 0.673 | 0.522 CI 95% [0.117, 0.928]  p = 0.012* |

*p value<0.05,

1. Z score is the mean of the Z scores of Digit Symbol Substitution Test, Category Fluency, Free and Cued Selective Reminding test (2)Mini-Mental State Examination (Orientation), it has been multiplied by 100 due to the small scale of weather effects
2. Z score multiplied by 100
3. Free and total recall
4. 1 to 100 VAS asking “How well does your memory works”
