# Supplementary material for: How weather affects cognitive and physical outcomes in older adults
Source: PLoS One. 2025 Nov 25;20(11):e0335866. doi: 10.1371/journal.pone.0335866 (PMC12646423; doi:10.1371/journal.pone.0335866)
Supplement: S6 Table — (DOCX) [file pone.0335866.s006.docx]

**Supplementary table 6: Effects of the weather (With a 10 days lag) on the probability to have an abnormally low scores (cognitive outcomes):**

|  | Composite Z-score (1)  N=812 (11.8%) | Digit Symbol Substitution Test  N=269 (3.9%) | Category Fluency  N=535 (7.75%) | Free and Cued Selective Reminding test (2)  N=1260 (18.3%) | Mini-Mental State Examination (Total) (3)  N=80 (1.16%) | Subjective memory performance (4)  N=108 (1.57%) |
| --- | --- | --- | --- | --- | --- | --- |
| *Temperature C° (for 10°C)* | | | | | | |
| Minimum | OR 1.05 (0.93,1.18)  p =0.442 | OR 1.07 (0.88,1.3)  p =0.476 | OR 1.19 (1.03,1.36)  p =0.017* | OR 1.07 (0.97,1.18)  p =0.154 | OR 0.86 (0.61,1.23)  p =0.416 | OR 0.66 (0.48,0.89)  p =0.007* |
| Mean | OR 1.03 (0.92,1.15)  p =0.642 | OR 1.11 (0.92,1.35)  p =0.276 | OR 1.19 (1.04,1.37)  p =0.013* | OR 1.07 (0.97,1.18)  p =0.156 | OR 0.93 (0.66,1.32)  p =0.686 | OR 0.71 (0.52,0.96)  p =0.025* |
| Maximum | OR 1.01 (0.92,1.12)  p =0.811 | OR 1.07 (0.91,1.27)  p =0.415 | OR 1.15 (1.02,1.3)  p =0.02* | OR 1.09 (1,1.19)  p =0.046* | OR 0.94 (0.69,1.28)  p =0.701 | OR 0.82 (0.63,1.07)  p =0.144 |
| *Humidex (for 10 points)* | | | | | | |
| Minimum | OR 1.04 (0.95,1.14)  p =0.426 | OR 1.05 (0.9,1.22)  p =0.559 | OR 1.14 (1.02,1.27)  p =0.02* | OR 1.07 (0.99,1.15)  p =0.098 | OR 0.91 (0.69,1.21)  p =0.528 | OR 0.73 (0.57,0.93)  p =0.011* |
| Mean | OR 1.02 (0.93,1.12)  p =0.655 | OR 1.06 (0.91,1.23)  p =0.435 | OR 1.14 (1.02,1.27)  p =0.016* | OR 1.06 (0.98,1.14)  p =0.14 | OR 0.94 (0.72,1.24)  p =0.678 | OR 0.76 (0.6,0.97)  p =0.025* |
| Maximum | OR 1.02 (0.93,1.1)  p =0.713 | OR 1.03 (0.89,1.18)  p =0.687 | OR 1.13 (1.02,1.25)  p =0.02* | OR 1.08 (1.01,1.16)  p =0.033* | OR 0.95 (0.73,1.22)  p =0.663 | OR 0.83 (0.66,1.03)  p =0.098 |

OR: Odd ratio, N : number of outliers, the total number of patients’ visits is 6900, *p value<0.05,

1. Z score is the mean of the Z scores of Digit Symbol Substitution Test, Category Fluency, Free and Cued Selective Reminding test (2)Mini-Mental State Examination (Orientation), an observation is considered abnormally low if it’s inferior of 0.3 SD or more than expected, 0.3S SD being the minimal clinically important difference.
2. An observation is considered abnormally low if it’s inferior by 6 points or more than expected, 6 points being the minimal clinically important difference.
3. An observation is considered abnormally low if it’s inferior by 5.15 points or more than expected, 5.15 points being the minimal detectable change.
4. Free and total recall, an observation is considered abnormally low if it’s inferior by 1.21 points or more than expected
5. MMSe total, an observation is considered abnormally low if it’s inferior by 3 points or more than expected, 3 points being the minimal clinically important difference.
6. 1 to 100 VAS asking “How well does your memory works”, An observation is considered abnormally low if it’s inferior by 24.9 points or more than expected, 24.9 points being the minimal detectable change
