# Supplementary material for: How weather affects cognitive and physical outcomes in older adults
Source: PLoS One. 2025 Nov 25;20(11):e0335866. doi: 10.1371/journal.pone.0335866 (PMC12646423; doi:10.1371/journal.pone.0335866)
Supplement: S8 Table — (DOCX) [file pone.0335866.s008.docx]

**Supplementary table 8: Effect of the weather on physical and functional outcomes**

|  | Gait Speed (m/s) | ADCS-ADL | Short Physical Performance Battery (SPPB) | Time to raise from chair (s) | Balance (0-4) | Hand Strength (kg) |
| --- | --- | --- | --- | --- | --- | --- |
| *Season (Reference Autumn)* | | | | | | |
| Spring | 0.00167 CI 95% [-0.0114, 0.0148]  p = 0.803 | 0.0266 CI 95% [-0.201, 0.254]  p = 0.818 | 0.116 CI 95% [0.0263, 0.207]  p = 0.011* | -0.123 CI 95% [-0.349, 0.102]  p = 0.283 | 0.0534 CI 95% [0.00852, 0.0983]  p = 0.02* | -0.164 CI 95% [-0.475, 0.147]  p = 0.302 |
| Summer | -0.0177 CI 95% [-0.0342, -0.0012]  p = 0.036* | -0.0568 CI 95% [-0.348, 0.234]  p = 0.702 | -0.0172 CI 95% [-0.131, 0.0968]  p = 0.767 | -0.00816 CI 95% [-0.293, 0.277]  p = 0.955 | 0.00279 CI 95% [-0.0514, 0.057]  p = 0.92 | 0.0297 CI 95% [-0.376, 0.435]  p = 0.886 |
| Winter | 0.0254 CI 95% [0.0104, 0.0405]  p = 0.001* | 0.108 CI 95% [-0.16, 0.375]  p = 0.431 | 0.163 CI 95% [0.0589, 0.268]  p = 0.002* | -0.247 CI 95% [-0.506, 0.0132]  p = 0.063 | 0.0731 CI 95% [0.0239, 0.122]  p = 0.004* | 0.0471 CI 95% [-0.324, 0.418]  p = 0.804 |
| *Temperature C° (for 10°C)* | | | | | | |
| Minimum | -0.017 CI 95% [-0.0244, -0.00974]  p = <0.001* | -0.0325 CI 95% [-0.16, 0.0947]  p = 0.616 | -0.0734 CI 95% [-0.124, -0.0229]  p = 0.004* | 0.0815 CI 95% [-0.0447, 0.208]  p = 0.206 | -0.0437 CI 95% [-0.069, -0.0185]  p = 0.001* | -0.122 CI 95% [-0.302, 0.0574]  p = 0.182 |
| Mean | -0.016 CI 95% [-0.0228, -0.00908]  p = <0.001* | -0.0511 CI 95% [-0.171, 0.0687]  p = 0.403 | -0.078 CI 95% [-0.125, -0.0305]  p = 0.001* | 0.0938 CI 95% [-0.0246, 0.212]  p = 0.121 | -0.045 CI 95% [-0.0688, -0.0213]  p = <0.001* | -0.156 CI 95% [-0.324, 0.0125]  p = 0.07 |
| Maximum | -0.0136 CI 95% [-0.0197, -0.00754]  p = <0.001* | -0.0486 CI 95% [-0.155, 0.0577]  p = 0.37 | -0.0724 CI 95% [-0.115, -0.0303]  p = 0.001* | 0.0819 CI 95% [-0.023, 0.187]  p = 0.126 | -0.04 CI 95% [-0.0611, -0.0189]  p = <0.001* | -0.157 CI 95% [-0.306, -0.00777]  p = 0.039* |
| *Humidex (for 10 points)* | | | | | | |
| Minimum | -0.0129 CI 95% [-0.0183, -0.00743]  p = <0.001 | -0.0309 CI 95% [-0.126, 0.0637]  p = 0.522 | -0.0565 CI 95% [-0.0941, -0.0189]  p = 0.003* | 0.062 CI 95% [-0.032, 0.156]  p = 0.196 | -0.0325 CI 95% [-0.0513, -0.0137]  p = 0.001* | -0.102 CI 95% [-0.235, 0.032]  p = 0.136 |
| Mean | -0.0123 CI 95% [-0.0175, -0.00706]  p = <0.001 | -0.0447 CI 95% [-0.136, 0.0465]  p = 0.337 | -0.059 CI 95% [-0.0951, -0.0228]  p = 0.001* | 0.0681 CI 95% [-0.0222, 0.158]  p = 0.139 | -0.0339 CI 95% [-0.0521, -0.0158]  p = <0.001* | -0.122 CI 95% [-0.25, 0.00641]  p = 0.063 |
| Maximum | -0.0113 CI 95% [-0.0162, -0.00637]  p = <0.001 | -0.0456 CI 95% [-0.131, 0.0398]  p = 0.295 | -0.0594 CI 95% [-0.0933, -0.0255]  p = 0.001* | 0.0614 CI 95% [-0.0231, 0.146]  p = 0.154 | -0.0328 CI 95% [-0.0497, -0.0158]  p = <0.001* | -0.122 CI 95% [-0.242, -0.00267]  p = 0.045* |

*p value<0.05
