# Supplementary material for: How weather affects cognitive and physical outcomes in older adults
Source: PLoS One. 2025 Nov 25;20(11):e0335866. doi: 10.1371/journal.pone.0335866 (PMC12646423; doi:10.1371/journal.pone.0335866)
Supplement: S9 Table — (DOCX) [file pone.0335866.s009.docx]

**Supplementary Table 9: Effect of the weather ((with 10 days lag) on physical and functional outcomes**

|  | Gait Speed (m/s) | ADCS-ADL | Short Physical Performance Battery (SPPB) | Time to raise from chair (s) | Balance (0-4) | Hand Strength (kg) |
| --- | --- | --- | --- | --- | --- | --- |
| *Temperature C° (for 10°C)* | | | | | | |
| Minimum | -0.0185 CI 95% [-0.0261, -0.011]  p = <0.001* | -0.0382 CI 95% [-0.17, 0.0936]  p = 0.57 | -0.0989 CI 95% [-0.151, -0.0466]  p = <0.001* | 0.148 CI 95% [0.0173, 0.279]  p = 0.026* | -0.0463 CI 95% [-0.0725, -0.0202]  p = 0.001* | -0.197 CI 95% [-0.38, -0.0147]  p = 0.034* |
| Mean | -0.0192 CI 95% [-0.0267, -0.0116]  p = <0.001* | -0.0403 CI 95% [-0.172, 0.0917]  p = 0.55 | -0.0896 CI 95% [-0.142, -0.0374]  p = 0.001* | 0.129 CI 95% [-0.00117, 0.26]  p = 0.052* | -0.0431 CI 95% [-0.0692, -0.017]  p = 0.001* | -0.0984 CI 95% [-0.281, 0.0841]  p = 0.291 |
| Maximum | -0.0159 CI 95% [-0.0224, -0.00938]  p = <0.001* | -0.0386 CI 95% [-0.153, 0.0755]  p = 0.507 | -0.078 CI 95% [-0.123, -0.0329]  p = 0.001* | 0.0888 CI 95% [-0.0237, 0.201]  p = 0.122 | -0.0381 CI 95% [-0.0606, -0.0155]  p = 0.001* | -0.0203 CI 95% [-0.178, 0.137]  p = 0.801 |
| *Humidex (for 10 points)* | | | | | | |
| Minimum | -0.0139 CI 95% [-0.0198, -0.0079]  p = <0.001* | -0.0394 CI 95% [-0.143, 0.0644]  p = 0.456 | -0.0784 CI 95% [-0.12, -0.0373]  p = <0.001* | 0.119 CI 95% [0.0167, 0.222]  p = 0.023* | -0.0362 CI 95% [-0.0568, -0.0156]  p = 0.001* | -0.143 CI 95% [-0.287, 0.00115]  p = 0.052 |
| Mean | -0.0141 CI 95% [-0.0199, -0.00832]  p = <0.001* | -0.03 CI 95% [-0.131, 0.0708]  p = 0.56 | -0.0697 CI 95% [-0.11, -0.0298]  p = 0.001* | 0.1 CI 95% [0.000611, 0.2]  p = 0.049 | -0.0336 CI 95% [-0.0535, -0.0136]  p = 0.001* | -0.0848 CI 95% [-0.224, 0.0547]  p = 0.234 |
| Maximum | -0.0137 CI 95% [-0.0192, -0.00827]  p = <0.001* | -0.0177 CI 95% [-0.113, 0.0774]  p = 0.715 | -0.0659 CI 95% [-0.103, -0.0283]  p = 0.001* | 0.0711 CI 95% [-0.0227, 0.165]  p = 0.138 | -0.0328 CI 95% [-0.0516, -0.014]  p = 0.001* | -0.0324 CI 95% [-0.164, 0.0988]  p = 0.628 |
