# Supplementary material for: How weather affects cognitive and physical outcomes in older adults
Source: PLoS One. 2025 Nov 25;20(11):e0335866. doi: 10.1371/journal.pone.0335866 (PMC12646423; doi:10.1371/journal.pone.0335866)
Supplement: S11 Table — (DOCX) [file pone.0335866.s011.docx]

**Supplementary table 11: Effects of the weather (with a 10 days lag) on the probability to have an abnormally low scores (functional and physical outcomes):**

|  | Gait Speed (m/s) (1)  N=2311 (34.1%) | ADCS-ADL (2)  N=138 (2.02%) | Short Physical Performance Battery (SPPB) (3)  N = 1363 (20.2%) | Time to raise from chair (s) (4)  N =124 (1.89%) | Balance (0-4) (5)  N = 238 (3.48%) | Hand Strength (kg) (6)  N = 140 (2.17%) |
| --- | --- | --- | --- | --- | --- | --- |
| *Temperature C° (for 10°C)* | | | | | | |
| Minimum | OR 1.17 (1.08,1.26)  p =< 0.001* | OR 0.97 (0.74,1.27)  p =0.818 | OR 1.15 (1.05,1.27)  p =0.003* | OR 1.08 (0.81,1.42)  p =0.614 | OR 1.11 (0.9,1.36)  p =0.32 | OR 1.02 (0.78,1.33)  p =0.888 |
| Mean | OR 1.11 (1.03,1.2)  p =0.01* | OR 0.88 (0.67,1.15)  p =0.344 | OR 1.15 (1.05,1.26)  p =0.004* | OR 1.05 (0.79,1.39)  p =0.729 | OR 1.1 (0.9,1.35)  p =0.352 | OR 0.88 (0.68,1.15)  p =0.351 |
| Maximum | OR 1.06 (0.99,1.13)  p =0.109* | OR 0.89 (0.7,1.12)  p =0.321 | OR 1.13 (1.04,1.23)  p =0.003* | OR 1.05 (0.82,1.34)  p =0.704 | OR 1.13 (0.94,1.35)  p =0.184 | OR 0.72 (0.57,0.91)  p =0.005* |
| *Humidex (for 10 points)* | | | | | | |
| Minimum | OR 1.12 (1.05,1.19)  p =< 0.001* | OR 0.97 (0.78,1.2)  p =0.784 | OR 1.13 (1.04,1.21)  p =0.002* | OR 1.07 (0.86,1.34)  p =0.544 | OR 1.1 (0.93,1.29)  p =0.268 | OR 0.99 (0.8017,1.2226)  p =0.926 |
| Mean | OR 1.08 (1.01,1.14)  p =0.017* | OR 0.9 (0.73,1.1)  p =0.299 | OR 1.12 (1.04,1.2)  p =0.003* | OR 1.05 (0.85,1.31)  p =0.633 | OR 1.09 (0.93,1.27)  p =0.3 | OR 0.91 (0.74,1.12)  p =0.376 |
| Maximum | OR 1.05 (0.99,1.11)  p =0.099 | OR 0.88 (0.72,1.07)  p =0.185 | OR 1.11 (1.04,1.19)  p =0.002* | OR 1.05 (0.85,1.28)  p =0.667 | OR 1.11 (0.95,1.28)  p =0.183 | OR 0.79 (0.65,0.96)  p =0.019* |

OR: Odd ratio, N : number of outliers, the total number of patients’ visits is 6900, *p value<0.05,

1. An observation is considered abnormally low if it’s inferior of 0.05 m/s or more than expected, 0.05m/s being the minimal clinically important difference.
2. An observation is considered abnormally low if it’s inferior of 5.58 points or more than expected, 5.58 points being the minimal detectable change.
3. An observation is considered abnormally low if it’s inferior by 0.55 point or more than expected, 0.55 points being the minimal clinically important difference.
4. An observation is considered abnormally low if it’s superior by 5.1 seconds or more than expected, 5.1 seconds being the minimal clinically important difference.
5. An observation is considered abnormally low if it’s inferior by 0.63 points or more than expected, 0.63 points being the minimal detectable change.
6. An observation is considered abnormally low if it’s inferior by 6.5 kg or more than expected, 6.5kg being the minimal clinically important difference.
