# Supplementary material for: How weather affects cognitive and physical outcomes in older adults
Source: PLoS One. 2025 Nov 25;20(11):e0335866. doi: 10.1371/journal.pone.0335866 (PMC12646423; doi:10.1371/journal.pone.0335866)
Supplement: S12 Table — (DOCX) [file pone.0335866.s012.docx]

**Supplementary table 12: Effects of the weather on the probability to have an abnormally low scores (functional and physical outcomes) using an alternative definition of abnormally:**

|  | Gait Speed (m/s) (1)  N=2799 (41.3%) | ADCS-ADL (2)  N=695 (10.2%) | Short Physical Performance Battery (SPPB) (3)  N = 1935 (28.66%) | Time to raise from chair (s) (4)  N =460 (6.99%) | Balance (0-4) (5)  N = 711 (10.4%) | Hand Strength (kg) (6)  N = 337 (5.23%) |
| --- | --- | --- | --- | --- | --- | --- |
| *Season (Reference Fall)* | | | | | | |
| Spring | OR 1.04 (0.91,1.19)  p =0.575 | OR 0.88 (0.7,1.09)  p =0.235 | OR 0.85 (0.73,0.98)  p =0.024* | OR 0.92 (0.71,1.19)  p =0.535 | OR 0.73 (0.59,0.9)  p =0.003* | OR 1.39 (1.02,1.89)  p =0.036* |
| Summer | OR 1.18 (1.02,1.36)  p =0.026* | OR 1.07 (0.85,1.35)  p =0.548 | OR 1.01 (0.87,1.18)  p =0.867 | OR 0.94 (0.71,1.25)  p =0.686 | OR 0.81 (0.65,1.02)  p =0.076 | OR 1.03 (0.71,1.48)  p =0.878 |
| Winter | OR 0.82 (0.72,0.93)  p =0.002* | OR 0.95 (0.77,1.17)  p =0.618 | OR 0.74 (0.64,0.85)  p =< 0.001* | OR 0.85 (0.66,1.09)  p =0.202 | OR 0.72 (0.59,0.88)  p =0.002* | OR 1.47 (1.09,1.97)  p =0.011* |
| *Temperature C° (for 10°C)* | | | | | | |
| Minimum | OR 1.18 (1.09,1.27)  p =< 0.001* | OR 1.06 (0.94,1.2)  p =0.361 | OR 1.13 (1.04,1.23)  p =0.004* | OR 0.92 (0.79,1.07)  p =0.29 | OR 1.1 (0.97,1.24)  p =0.133 | OR 1.07 (0.9,1.28)  p =0.426 |
| Mean | OR 1.14 (1.06,1.23)  p =< 0.001* | OR 1.07 (0.95,1.2)  p =0.272 | OR 1.14 (1.05,1.23)  p =0.001* | OR 0.98 (0.85,1.12)  p =0.734 | OR 1.09 (0.97,1.22)  p =0.161 | OR 1.05 (0.9,1.24)  p =0.523 |
| Maximum | OR 1.1 (1.04,1.18)  p =0.002* | OR 1.06 (0.95,1.17)  p =0.289 | OR 1.12 (1.05,1.2)  p =< 0.001* | OR 0.9989 (0.8826,1.1305)  p =0.986 | OR 1.07 (0.96,1.18)  p =0.222 | OR 1.02 (0.89,1.18)  p =0.746 |
| *Humidex (for 10 points)* | | | | | | |
| Minimum | OR 1.13 (1.06,1.19)  p =< 0.001* | OR 1.05 (0.96,1.15)  p =0.299 | OR 1.1 (1.03,1.17)  p =0.003* | OR 0.94 (0.84,1.05)  p =0.288 | OR 1.07 (0.98,1.17)  p =0.151 | OR 1.04 (0.91,1.18)  p =0.595 |
| Mean | OR 1.11 (1.05,1.17)  p =< 0.001* | OR 1.06 (0.97,1.16)  p =0.204 | OR 1.1 (1.04,1.17)  p =0.001* | OR 0.97 (0.88,1.08)  p =0.631 | OR 1.07 (0.98,1.16)  p =0.155 | OR 1.02 (0.91,1.16)  p =0.708 |
| Maximum | OR 1.09 (1.03,1.15)  p =0.001* | OR 1.06 (0.97,1.15)  p =0.193 | OR 1.11 (1.05,1.17)  p =< 0.001* | OR 0.99 (0.89,1.09)  p =0.817 | OR 1.06 (0.97,1.15)  p =0.182 | OR 1.01 (0.9,1.13)  p =0.864 |

OR: Odd ratio, N : number of outliers, the total number of patients’ visits is 6900, *p value<0.05,

1. An observation is considered abnormally low if it’s inferior of 0.03 m/s or more than expected, 0.03m/s being the lower limit of the minimal clinically important difference estimation.
2. An observation is considered abnormally low if it’s inferior by 2.84 point or more than expected, 2.84 being the standard error of measurement
3. An observation is considered abnormally low if it’s inferior by 0.27 point or more than expected, 0.27 points being the lower limit of the minimal clinically important difference estimation.
4. An observation is considered abnormally low if it’s superior by 2.6 seconds or more than expected, 2.6 seconds being the lower limit of the minimal clinically important difference estimation.
5. An observation is considered abnormally low if it’s inferior by 1.24 point or more than expected, 1.24 being the standard error of measurement
6. An observation is considered abnormally low if it’s inferior by 5 kg or more than expected, 5kg being the lower limit of the minimal clinically important difference estimation.
