# Supplementary material for: How weather affects cognitive and physical outcomes in older adults
Source: PLoS One. 2025 Nov 25;20(11):e0335866. doi: 10.1371/journal.pone.0335866 (PMC12646423; doi:10.1371/journal.pone.0335866)
Supplement: Attachment — Submitted filename: Response to reviewers.docx [file pone.0335866.s013.docx]

*Journal Requirements:*

*When submitting your revision, we need you to address these additional requirements.*

*1. Please ensure that your manuscript meets PLOS ONE's style requirements, including those for file naming. The PLOS ONE style templates can be found at*

[*https://journals.plos.org/plosone/s/file?id=wjVg/PLOSOne_formatting_sample_main_body.pdf*](https://journals.plos.org/plosone/s/file?id=wjVg/PLOSOne_formatting_sample_main_body.pdf) *and*

[*https://journals.plos.org/plosone/s/file?id=ba62/PLOSOne_formatting_sample_title_authors_affiliations.pdf*](https://track.editorialmanager.com/CL0/https:%2F%2Fjournals.plos.org%2Fplosone%2Fs%2Ffile%3Fid=ba62%2FPLOSOne_formatting_sample_title_authors_affiliations.pdf/1/010f0196c39e210f-38539dce-cbb9-41bc-9555-6bbdb0cd84ef-000000/OyFSygIa_XiwLj0Ohd_TKq-pqJvYD0_2Q1Ag_dLL688=210)*.*

The manuscript has been modified accordingly.

*2. We note that you have indicated that there are restrictions to data sharing for this study. For studies involving human research participant data or other sensitive data, we encourage authors to share de-identified or anonymized data. However, when data cannot be publicly shared for ethical reasons, we allow authors to make their data sets available upon request. For information on unacceptable data access restrictions, please see* [*http://journals.plos.org/plosone/s/data-availability#loc-unacceptable-data-access-restrictions*](http://journals.plos.org/plosone/s/data-availability#loc-unacceptable-data-access-restrictions)*.*

*Before we proceed with your manuscript, please address the following prompts:*

*a) If there are ethical or legal restrictions on sharing a de-identified data set, please explain them in detail (e.g., data contain potentially identifying or sensitive patient information, data are owned by a third-party organization, etc.) and who has imposed them (e.g., a Research Ethics Committee or Institutional Review Board, etc.). Please also provide contact information for a data access committee, ethics committee, or other institutional body to which data requests may be sent.*

*b) If there are no restrictions, please upload the minimal anonymized data set necessary to replicate your study findings to a stable, public repository and provide us with the relevant URLs, DOIs, or accession numbers. Please see* [*http://www.bmj.com/content/340/bmj.c181.long*](http://www.bmj.com/content/340/bmj.c181.long) *for guidelines on how to de-identify and prepare clinical data for publication. For a list of recommended repositories, please see* [*https://journals.plos.org/plosone/s/recommended-repositories*](https://journals.plos.org/plosone/s/recommended-repositories)*. You also have the option of uploading the data as Supporting Information files, but we would recommend depositing data directly to a data repository if possible.*

*Please update your Data Availability statement in the submission form accordingly.*

Although Nicola Coley is a member of the MAPT DSA group, the authors do not have individual permission to share the data. The datasets generated and/or analyzed during this study are not publicly available, and have to be requested from the MAPT DSA group. However, individual de-identified participant data are available with immediate effect to academic researchers, following approval of a methodologically sound research proposal by the MAPT data sharing committee and signature of a data use agreement. Enquiries or proposals should be directed to [nicola.coley@inserm.fr](mailto:nicola.coley@inserm.fr) and [guyonnet.s@chu-toulouse.fr](mailto:guyonnet.s@chu-toulouse.fr).

*3. Thank you for stating the following in the Acknowledgments Section of your manuscript:*

*“The MAPT study was supported by grants from the Gérontopôle of Toulouse, the French Ministry of Health (PHRC 2008, 2009), Pierre Fabre Research Institute (manufacturer of the omega-3 supplement), ExonHit Therapeutics SA, and Avid Radiopharmaceuticals Inc. The promotion of this study was supported by the University Hospital Centre of Toulouse. The data sharing activity was supported by the Association Monegasque pour la Recherche sur la maladie d’Alzheimer (AMPA) and the INSERM-University of Toulouse III UMR 1295 Research Unit (CERPOP). The IHU HealthAge Open Science initiative is supported by the French National Research Agency (ANR) as part of the France 2030 program (reference number: ANR-23-IAHU-0011).This secondary analysis didn’t receive any specific funding or grant”*

*We note that you have provided additional information within the Acknowledgements Section that is not currently declared in your Funding Statement. Please note that funding information should not appear in the Acknowledgments section or other areas of your manuscript. We will only publish funding information present in the Funding Statement section of the online submission form.*

*Please remove any funding-related text from the manuscript and let us know how you would like to update your Funding Statement. Currently, your Funding Statement reads as follows:*

*“The author(s) received no specific funding for this work.”*

*Please include your amended statements within your cover letter; we will change the online submission form on your behalf.*

Both statements are correct, the authors did not receive funding for this work, however the MAPT study itself for which we obtained the data has received the previously mentioned funding.

*4. One of the noted authors is a group or consortium [MAPT/IHU HealthAge Open Science study group#MAPT/DSA Study Group]. In addition to naming the author group, please list the individual authors and affiliations within this group in the acknowledgments section of your manuscript. Please also indicate clearly a lead author for this group along with a contact email address.*

Sorry, I don’t understand this request, The list was already present in the acknowledgment section.

*5. Your ethics statement should only appear in the Methods section of your manuscript. If your ethics statement is written in any section besides the Methods, please move it to the Methods section and delete it from any other section. Please ensure that your ethics statement is included in your manuscript, as the ethics statement entered into the online submission form will not be published alongside your manuscript.*

The manuscript has been modified accordingly

*6. Please include captions for your Supporting Information files at the end of your manuscript, and update any in-text citations to match accordingly. Please see our Supporting Information guidelines for more information:* [*http://journals.plos.org/plosone/s/supporting-information*](http://track.editorialmanager.com/CL0/http:%2F%2Fjournals.plos.org%2Fplosone%2Fs%2Fsupporting-information/1/010f0196c39e210f-38539dce-cbb9-41bc-9555-6bbdb0cd84ef-000000/9e_ijCXnBohu4GorSlS_D5owjrI5xPV3T1ruIivMvl0=210)*.*

We included caption for all supplementary tables

*Reviewers' comments:

Reviewer's Responses to Questions*

***Comments to the Author***

*1. Is the manuscript technically sound, and do the data support the conclusions?

The manuscript must describe a technically sound piece of scientific research with data that supports the conclusions. Experiments must have been conducted rigorously, with appropriate controls, replication, and sample sizes. The conclusions must be drawn appropriately based on the data presented.*

*Reviewer #1: No*

*Reviewer #2: Yes*

*Reviewer #3: Partly*

*2. Has the statistical analysis been performed appropriately and rigorously?*

*Reviewer #1: Yes*

*Reviewer #2: Yes*

*Reviewer #3: I Don't Know*

*3. Have the authors made all data underlying the findings in their manuscript fully available?

The* [*PLOS Data policy*](http://track.editorialmanager.com/CL0/http:%2F%2Fwww.plosone.org%2Fstatic%2Fpolicies.action%23sharing/1/010f0196c39e210f-38539dce-cbb9-41bc-9555-6bbdb0cd84ef-000000/89cmF9O5FbcmByduU0B68zNKFCeTNp4T-nzwk_cUg0c=210) *requires authors to make all data underlying the findings described in their manuscript fully available without restriction, with rare exception (please refer to the Data Availability Statement in the manuscript PDF file). The data should be provided as part of the manuscript or its supporting information, or deposited to a public repository. For example, in addition to summary statistics, the data points behind means, medians and variance measures should be available. If there are restrictions on publicly sharing data—e.g. participant privacy or use of data from a third party—those must be specified.*

*Reviewer #1: Yes*

*Reviewer #2: No*

*Reviewer #3: No*

*4. Is the manuscript presented in an intelligible fashion and written in standard English?

PLOS ONE does not copyedit accepted manuscripts, so the language in submitted articles must be clear, correct, and unambiguous. Any typographical or grammatical errors should be corrected at revision, so please note any specific errors here.*

*Reviewer #1: Yes*

*Reviewer #2: Yes*

*Reviewer #3: Yes*

*5. Review Comments to the Author

Please use the space provided to explain your answers to the questions above. You may also include additional comments for the author, including concerns about dual publication, research ethics, or publication ethics. (Please upload your review as an attachment if it exceeds 20,000 characters)*

*Reviewer #1: I am glad to review the manuscript entitled 'How weather affects clinical outcomes in elderly subjects'. Althogh this is an interesting topic, the clinical implication and conclusion are quite limited. In this study, weather might affect the clinical outomes of elderly patients through the changes of temperature, but there are many influence factors. First of all, patients were not alwaya exposed to the outdoor condictions and indoor temperature is controllable. Sencodly, some emotional sensitive patents might be influenced by the wether, but such factors were not able to be calculated in this study.*

Indeed our study could not account for factors such as personal sensitivity or individual indoor, or in transport conditions that probably play a role in mediating weather effects on patients and further study could be performed. However, as we mentioned in the discussion section of the article “Although this is clearly a limitation in terms of the explanatory value of our models, it facilitates applicability. Indeed, simple aggregation techniques and observed meteorological data could be reasonably employed in clinical and research settings.”

Furthermore, this is a rather conservative limitation, meaning that although we might not show some association due to adaptive strategies such indoor climatisation, the associations that we showed are very unlikely to be caused by these adaptive strategies.

Therefore we updated the discussion paragraph (sentences underlined are new) in :”A second limitation is our use of simple aggregation techniques for the day’s weather (mean, minimum and maximum) or our lags, rather than more complex models^12^ and our exclusive use of external weather rather than precise modeling of each environment encountered by participants (including home and transportation) . Although this is clearly a limitation in terms of the explanatory value of our models, it facilitates applicability. Indeed, simple aggregation techniques and observed meteorological data could be reasonably employed in clinical and research settings. On the other hand, it could prove very difficult to model participants’ entire environment including indoor, transport and facility temperature and humidity. Furthermore, it is important to note that this limitation is conservative, meaning that although we might not show some associations the ones present are unlikely to be false positive. “

Reviewer #2: Dear Dr. Sittiprapaporn,
Thank you for the opportunity to review the manuscript entitled “How weather affects clinical outcomes in elderly subjects”.
The paper addresses an important and often overlooked aspect of geriatric assessment by exploring the impact of weather conditions on cognitive and physical performance in older adults. The findings, particularly regarding the influence of temperature on gait speed and physical function, have clear implications for both clinical practice and future research. I have provided comments and suggestions to enhance the clarity, depth, and rigor of the authors discussion and conclusions. I hope these recommendations will strengthen the manuscript and further its contribution to the field.
Thank you again for the opportunity to contribute to the review process.


*Title
1. The title is broad and appeal to a wide audience, however, the term “clinical outcomes” is vague, especially since the study focuses specifically on cognitive and physical performance.*

We changed the title for “How weather affects cognitive and physical outcomes in older adults”

2. “Elderly subjects” is outdated and less respectful language; current best practice prefers terms like “older adults”.

We changed the title for “How weather affects cognitive and physical outcomes in older adults”

*Abstract
3. Objective: The sentence is unclear and slightly awkward. "…in relation to the expected abilities…" lacks clarity. Please rephrase it.*

We changed the objective to” “To ascertain whether, in comparison to the participants' expected abilities, the weather may cause abnormally poor cognitive or physical performance.”

*4. Setting: Consider integrating "France" naturally.*

We changed the setting to “Study conducted between May, 2008, and Feb, 2011 in 13 memory centres in France and Monaco”

*5. Participants: Good description, but the inclusion criteria feel jammed into the sentence. Consider breaking into two sentences or using parentheses.*

We changed the participants section in : “1313 participants from the MAPT trial, a 5-year multicentre prevention trial, which included dementia-free individuals aged over 70 years. Participants presented subjective memory complaints, slow gait speed and/or an IADL limitation.”

*6. Outcomes and Methods
7. The definition of "abnormally low scores" is important but buried. Also, the abbreviation "SPPB" is used before being defined. Introduce abbreviations before use, and perhaps rephrase.*

The SPPB abbreviation was introduced in the main outcome section at the time of its first use (line 23 p7). We modified the abnormally low score definition as such: “Abnormally low scores were defined as an observed score that was lower than the individual's expected ability by at least the minimal clinical important difference.”

*8. Results: Generally, well presented with statistics but could be clearer on what outcomes were not significant.*

We changed the result section to: “Higher temperature was associated with a significantly increased risk of abnormally low gait speed or SPPB (respectively OR 1.13 95% CI (1.04, 1.22) and OR 1.15 95% CI (1.03, 1.29) for 10 degrees), but did not significantly increase the risk of abnormally low cognitive function”.

9. The structure of "Specifically, temperature also associated with..." is awkward.

This sentence was deleted.
10. Conclusion is reasonable and practical, but could emphasize the implication for clinical trials and practice more strongly.

We modified the conclusion to: “Our results suggest that weather conditions should be strongly considered when assessing the physical performance of older adults in the context of clinical practice and clinical research, as examination in hot weather might lead to false conclusions on the participants' abilities.”

*Introduction
The introduction presents a relevant and timely research question within the context of aging, climate change, and functional health assessment. However, the narrative is occasionally disjointed and would benefit from clearer transitions, refined phrasing, and improved organization of ideas. Some claims are underdeveloped or insufficiently supported.
11. Several sentences are awkwardly phrased or grammatically incorrect (e.g., “the concepts of healthy aging and intrinsic capacities… have shown their relevance…” is vague and unclear).*

This sentence has been rephrased as “The concepts of healthy aging and intrinsic capacities—which place more emphasis on function than diseases—have proven useful in forecasting the health, mortality, and quality of life of older persons in the setting of a rapidly aging population.”

*12. The logic behind the narrative jumps abruptly from methodological challenges in aging research to climate projections without smooth transitions.*

We deleted all mention of climate projection from the introduction.

13. The paragraph about IPCC projections interrupts the development of the argument about clinical heterogeneity.

See answer above, additionally weather projection and effects are necessary context to explain how and why they might play a role in heterogeneity.

*14. The concept of “abnormally low performance unreflective of actual function” is introduced late and could be foregrounded earlier.*

The rest of the elements are necessary context to introduce this notion, which, without the developments on heterogeneity and weather as a cause of heterogeneity would be difficult to comprehend.

*15. The difference between weather, temperature, and climate needs to be clearly defined early on.*

We added the following sentence : “However, weather, i.e. the state of the atmosphere at a given time and place, affects individuals throughout the day and in more complex ways than temperature during evaluation.”

*16. The introduction notes conflicting prior findings, but doesn’t explain what the current study does differently or why it fills a unique gap.*

We gave the following justification in the introduction : Furthermore, these studies did not try to determine whether the performance of an individual could be unreflective of their actual function because of the weather at the time it was evaluated.

*17. Citations are densely packed and not always well integrated into the argument (e.g., references 11–16 are dropped at the end of a sentence without context).*

In line with standard practice, we kept our introduction relatively brief and did not present these studies at great length. However, there is a more detailed discussion of their results in the discussion section.

*Methods:
18. Consider briefly stating the rationale for including individuals with subjective memory complaints, prefrailty, or slow gait to contextualize their relevance to cognition research.*

This study reuses the data from the MAPT trial. The rationale for the inclusion criteria is explained in Gillette-Guyonnet S et al. Alzheimers Dement. 2009;5(2):114-21, PMID: 19328438. Briefly, the rationale was that frailty was considered to be a risk factor for cognitive decline and dementia in older people. At the time of the study, there was no consensus about the definition of frailty, and so it was decided to use three criteria thought to reflect cognitive and/or physical frailty, and to target individuals towards the “pre-frail” end of the frailty spectrum.

We have added the following sentence to the methods section to clarify this point: "These criteria were chosen to identify people who are believed to be at a higher risk of cognitive decline or dementia and who are in the early stages of cognitive and/or physical frailty." and the following reference : Gillette-Guyonnet S et al. Alzheimers Dement. 2009;5(2):114-21, PMID: 19328438

19. The intervention groups are well-described. However, the structure could be cleaner.
Consider bulleting or rephrasing for clarity

We bulleted the intervention groups

*20. Good contextual reference to the MAPT-plus follow-up and participant characteristics. Clarify “without normalization” and explain why individual test scores were also analyzed this way.*

We clarified as follows: “without normalization i.e Z-score transformation, as this is the way they would be used in clinical practice”

We added the following sentence :
*Outcomes
21. Cognitive Outcomes: Clarify that the Z-scores were standardized based on baseline means/SDs.*

We added the following sentence : “As in the MAPT trial, the Z score was calculated using baseline means and standard deviation.”

*Also, state the rationale for expressing Z-scores as percentages (multiplied by 100)—was it for easier interpretation?*

Indeed, please refer to the sentence “To facilitate readability, the composite Z-score is presented as a percentage (i.e. multiplied by 100).”

*22. Functional/Physical Outcomes: For clarity, briefly mention the meaning or significance of the SPPB and ADCS-ADL-PI scores, especially for readers unfamiliar with these instruments.*

We added the following explanations : The SPPB is a global measure of physical capacity, and mobility which comprises three evaluations (Gait Speed, Time to rise from chair and balance) each scored on a 4 point scale. The ADCS-ADL-PI is a fifteen item questionnaire developed to assess early alterations of activities of daily living (like preparing meals or getting dressed).

*23. Explanatory Variables: Consider adding a sentence on the relevance of weather to cognitive and physical outcomes in aging populations (e.g., thermal stress, reduced activity levels).*

The aim of this article is to evaluate the link between weather and these performances. The justification for performing this study therefore lies mainly in the introduction with sentences such as : “This could make heat waves up to 12 times more frequent. These extreme events have a well-known effect on morbidity and mortality, especially amongst older adults”

*24. Descriptive and Inferential Approach:
A clearer explanation can improve the phrase “time, time², time³” (e.g., “modeled time as a cubic polynomial”) for a more general audience.*

We modified the sentence as suggested: “Initial models included: participant-specific random slopes and intercepts, the fixed effects of time modeled time as a cubic polynomial, age and its interaction with time polynomials, sex, level of education and centre”

*25. The logic behind using a 30% SD cutoff for the FCSRT is explained; do the other MCIDs also correspond to clinically meaningful changes in older adults? If so, briefly cite this rationale.*

We added the following sentence : Indeed the minimal important clinical difference (MCID represents clinical meaningful change, which could therefore lead to clinical consequences (decision not to treat).

*26. State why only bivariate models were used for weather-abnormal scores analysis. Would adjusting for baseline characteristics strengthen the findings?*

We thank the reviewer for this remark. However, abnormally scores are defined from residuals of the model including baseline characteristics. In linear mixed models models, residuals are independent from explanatory variables. For more clarity we added the following sentence : “Because residuals are independent from explanatory variables we did not include age, sex or education level in the abnormally low score analysis.”

*27. Briefly comment on the proportion of missing data for key variables, if available.*

As mentioned in the flowchart, 31 patients had a missing value for level of education. As mentioned in line 11 p 10, using a linear mixed model, means there are no other missing values for participants as slopes are modelled on all available visits using a continuous time scale. There was no day without any meteorological data.

*28. Not adjusting for multiple comparisons is acceptable for exploratory work, but this should be acknowledged as a limitation, either here or in the Discussion.*

Yes indeed, please refer to the following sentence: “Due to the exploratory nature of this analysis we did not adjust for multiple tests. “

*29. Improve clarity and readability in complex sentences (e.g., polynomial modeling, random slopes).*

We tried to simplify all complex sentences. However, some technical terms, such as random slopes, are difficult to avoid when using this type of model.

*30. Justify methodological decisions in more reader-friendly language (e.g., Z-score % scaling, bivariate regressions).*

As formulated above, we tried to simplify all complex sentences. However some technical terms are difficult to avoid when using these types of models.

*31. Minor rewording for consistency and grammar (e.g., “made accessed” → “were accessed”).*

We modified this to: “made accessible” *Results
This results section is strong in terms of content and statistical clarity. With some adjustments in language, transitions, and layout, it could be more accessible to a broader audience while retaining its scientific rigor.
32. The section is rich in data but better flow and transitions between parts can improve it.*

We tried to follow standard practice when presenting our results, i.e. a very factual presentation without discussion or interpretation. Thus it may seem that there is a lack of transitions between parts, but we consider this to be standard for results sections.

*33. Repetitions like "respectively" and multiple numbers in one sentence can overwhelm the reader.*

We only kept formulation including respectively when presenting results for minimum, mean and maximum temperature where it would be very tedious to repeat entire sentences.

*34. It might help to group related findings more clearly, especially for readers not familiar with all measures.*

We chose to group findings by type of function effect of season or temperature and direct effect or effect on abnormally low score. Were there other elements that results should be grouped as in your opinion?

*35. The main findings are present but readability is slightly hindered by stats-heavy phrasing.
36. Sensitivity analyses with 3- and 10-day lags confirmed the main findings: higher temperatures and humidex were associated with poorer FCSRT scores and improved subjective memory (see Supplementary Tables 3 and 4).
37. Seasonality and Abnormally Low Cognitive Performance is well presented but could use minor structural improvements.
38. Physical/Functional Outcomes – Main Effects are well structured but
39. Physical Measures & Temperature include good detail but some stat-heavy phrasing.
40. Temperature & Abnormally Low Physical Scores are technically sound, just tidy phrasing.*

We endeavoured to make the results section as readable as possible, but we believe that some mention of numerical results is necessary. Could you expand or be more specific in what you considered stats-heavy phrasing or which sentence needs rephrasing ?

Discussion
*41. Currently, the discussion is a bit dense and jumps quickly between ideas. Consider reorganizing it into clearer sub-sections or paragraphs:
• Summary of main findings
• Comparison with literature
• Methodological strengths and limitations
• Interpretation of differential effects
• Implications and future directions*

We added the recommended subheading, but left future directions for the conclusion

*42. The discussion suggests that weather might influence assessment outcomes, especially gait speed. Emphasize how this could affect clinical trials, screening programs, or routine geriatric evaluations.*

We added the following paragraph : “Correct assessment of physical capacities, including gait speed is of prime importance in clinical trials or geriatric evaluations. In clinical trials gait speed can be used as an inclusion criterion or as an outcome. In the context of inclusion criteria, not accounting for weather could therefore bar some participants from receiving the intervention and introduce selection bias. Regarding gait speed as an outcome, ignoring weather could lead to measurement bias and loss of power. In the context of routine geriatric evaluations, underevaluating patients’ capacity could bar them from receiving important treatments like invasive surgery or chemotherapy.”

*43. Should clinicians adjust thresholds seasonally? Should testing environments be climate-controlled?*

These are very interesting follow-up questions from this study. Indeed, now that we have shown such an effect, clearly defining how to account for it is the next step. There is some limited literature on climate controlled assessment in young adults (Cedeño Laurent JG,et al Reduced cognitive function during a heat wave among residents of non-air-conditioned buildings: An observational study of young adults in the summer of 2016. *PLoS Med*. 2018;) however this doesn’t account for most of the time spent in hot weather which is not during evaluation time but at home or on the way to the evaluation center. We definitely think that MCID thresholds and scores norms should be adapted depending on the weather and this will be the purpose of a future study. Therefore, we added the following sentence : “Due to the increased number of heatwaves, another approach would be to adapt norms and interpretation of decline (Minimal clinical Important difference) depending on the weather.”

*44. Address possible confounders: You discuss depressive symptoms and education as potential confounders in other studies. Consider also discussing whether mood, hydration, or transportation burden could confound your own findings, especially on test days.*

Rather than confounding factors these are mediating factors, indeed as mentioned in the discussion the effect of weather is plurifactorial. As suggested we mentioned mood, hydration, or transportation burden along with other mediating factors in the sentence : “Namely, we didn’t explore mechanisms and mediating factors such as mood, hydration, or transportation burden through which weather affects cognitive and physical function, and further studies are needed that could explain why one seems to be affected but not the other.”

*45. Tone down the certainty in some comparative statements. For example: "the association they found might have been exaggerated..." . Consider rephrasing to soften speculation unless supported by direct evidence.*

We replaced exaggerated with overestimated.
*Conclusion
46. Add a brief nod to public health implications. A sentence could be added about the importance of this knowledge in population aging, heat waves, or urban planning for older adults.*

We added the following sentence in conclusion : “Additionally the influence of weather on older adults should be accounted for in the design of medical facilities and city planning.”

*47. Rephrase "evaluations might need to be rescheduled if the weather is too hot,"*

We rephrased this sentence as : “Assessments may need to be rescheduled if the temperature is excessively high.”

*Reviewer #3: 1. This manuscript investigates whether outdoor weather might influence physical and cognitive performance of patients tested in an indoor clinical setting. When I read the abstract, I thought the authors were investigating temperature inside the clinic, and I wondered why their clinic has such variable temperatures. It became clear when reading the introduction that weather outside during the course of an entire day might be more important to a patient’s performance than the temperature of the clinic, which they may only experience briefly. This is a novel and interesting idea. The abstract needs to more clearly describe this approach.
2. Abstract: Define unit of measurement for temperature: Celsius.*

We defined the unit of measurement as suggested.

*3. I appreciate that the authors’ are taking a fresh look at archival data to answer a new question.*

Thanks a lot for your comment.

*4. Table 1: Not enough info is given about what the values mean. The legend/footnote should be expanded.*

We added the range and interpretation for each score and measure used.

*5. Figures 2 and 3 are so blurry I cannot read them.*

This is very unfortunate, The files appeared clear in the submission, but if a higher resolution is required by the editor, we would be happy to provide it.

*6. In the Results section “Effect on cognitive outcomes,” Table 3 is referenced, but there is no Table 3.*

We are very sorry for this mistake. It was actually Supplementary table 3 we were referring to. This has been corrected.

*7. I find the conclusion of this study intriguing, that outdoor weather should be considered even for indoor testing, and rescheduling may be valuable when weather is very hot. This could have an impact on clinical practice. However, I am unable to assess the validity of the conclusions because the figures are not legible, tables are not well described, and there is a missing table.*

We thank the reviewer for this positive assessment. We hope that our responses to the above comments resolve the issues relating to the figures and tables.
