# Supplementary material for: How weather affects cognitive and physical outcomes in older adults
Source: PLoS One. 2025 Nov 25;20(11):e0335866. doi: 10.1371/journal.pone.0335866 (PMC12646423; doi:10.1371/journal.pone.0335866)
Supplement: Attachment — Submitted filename: renamed_4aad7.docx [file pone.0335866.s014.docx]

Reviewer #4: This is a technically sound paper, the data support the conclusions, and the revisions adequately address most of the reviewers' comments. The conclusion that outdoor weather is associated with impaired physical performance in clinical settings is important and worthy of publication. One minor point: the introduction shows signs of having been written with the help of artificial intelligence, as long dashes in the sentence are usually used by AI, and the highlighting of individual words is also used by AI.

We thank the reviewer for this verification, but in fact we did not use any AI tools during the writing of the manuscript. We used a commonly available AI detection tool to try and change the aforementioned signs but unsurprisingly given that no AI was used, no AI was detected. Furthermore, to avoid confusion we deleted highlighting and long dashes.

Reviewer #5: I am honored for the opportunity to review your manuscript entitled “How weather affects clinical outcomes in elderly subjects.” This study addressed a unique perspective better understanding the health of elderly adults and how it is impacted by the weather. I have listed a few recommendations that address grammatical and punctuation errors as well as some suggestions to strengthen the clarity of the manuscript.

We thank the reviewer and have addressed all the suggested changes.

Introduction
Consider rephrasing ‘older persons’ to ‘elderly adults’ :

We have rephrased to “older adults”. We used “older” rather than “elderly” in light of the previous reviewer’s comments.

Consider removing ‘Indeed’ and going straight into ‘Targeting function…’ or ‘There are challenges faced when targeting function rather than…

We made the suggested change.

Consider changing “Because of’ to “Due to…”

We made the suggested change.

The sentence on line 21 is a bit odd, consider changing “was” to “with” and cutting out repeated words (mostly, studies/studying).

We changed the sentence to: “Furthermore, most previous work consisted of physiological studies of small groups of healthy young adults in highly controlled experiments, rather than everyday life^”^

Add a space after i.e (line 23).

We made the suggested change.

Discussion
Exclude “Our study has two main results.” and replace it with “The results from the study conclude that…”

We made the suggested change.

Remove extra space on line 7.

We made the suggested change.
